# Hospitalization and definitive radiotherapy in lung cancer: incidence, risk factors and survival impact

**DOI:** 10.1186/s12885-020-06843-z

**Published:** 2020-04-19

**Authors:** Sarah Z. Hazell, Nicholas Mai, Wei Fu, Chen Hu, Cole Friedes, Alex Negron, Khinh Ranh Voong, Josephine L. Feliciano, Peijin Han, Samantha Myers, Todd R. McNutt, Russell K. Hales

**Affiliations:** 1 Department of Radiation Oncology and Molecular Radiation Sciences, Johns Hopkins University School of Medicine, 300 Mason Lord Drive, Baltimore, MD 21224 USA; 2 Department of Oncology, Biostatistics, Johns Hopkins University School of Medicine, Baltimore, MD USA; 3 Department of Oncology, Johns Hopkins University School of Medicine, Baltimore, MD USA

**Keywords:** Lung cancer, Radiation, Hospitalization, Mortality, Nomogram

## Abstract

**Background:**

Unplanned hospitalization during cancer treatment is costly, can disrupt treatment, and affect patient quality of life. However, incidence and risks factors for hospitalization during lung cancer radiotherapy are not well characterized.

**Methods:**

Patients treated with definitive intent radiation (≥45 Gy) for lung cancer between 2008 and 2018 at a tertiary academic institution were identified. In addition to patient, tumor, and treatment related characteristics, specific baseline frailty markers (Charlson comorbidity index, ECOG, patient reported weight loss, BMI, hemoglobin, creatinine, albumin) were recorded. All cancer-related hospitalizations during or within 30 days of completing radiation were identified. Associations between baseline variables and any hospitalization, number of hospitalizations, and overall survival were identified using multivariable linear regression and multivariable Cox proportional-hazards models, respectively.

**Results:**

Of 270 patients included: median age was 66.6 years (31–88), 50.4% of patients were male (*n* = 136), 62% were Caucasian (*n* = 168). Cancer-related hospitalization incidence was 17% (*n* = 47), of which 21% of patients hospitalized (*n* = 10/47) had > 1 hospitalization. On multivariable analysis, each 1 g/dL baseline drop in albumin was associated with a 2.4 times higher risk of any hospitalization (95% confidence interval (CI) 1.2–5.0, *P* = 0.01), and baseline hemoglobin ≤10 was associated with, on average, 2.7 more hospitalizations than having pre-treatment hemoglobin > 10 (95% CI 1.3–5.4, *P* = 0.01). After controlling for baseline variables, cancer-related hospitalization was associated with 1.8 times increased risk of all-cause death (95% CI: 1.02–3.1, *P* = 0.04).

**Conclusions:**

Our data show baseline factors can predict those who may be at increased risk for hospitalization, which was independently associated with increased mortality. Taken together, these data support the need for developing further studies aimed at early and aggressive interventions to decrease hospitalizations during treatment.

## Background

Unplanned hospitalizations during definitive therapy for lung cancer are costly, can disrupt treatment, and affect patient quality of life. In a SEER database analysis, 60% of lung cancer patients receiving chemotherapy underwent an unplanned hospitalization with a mean of 1.5 hospitalizations per patient and a median Medicare charge of $31,036 per hospitalization [1]. Understanding the predictive factors associated with unplanned hospitalization during a course of definitive thoracic radiation (RT) can help identify those who may benefit most from close monitoring and early intervention.

Chemotherapy related toxicity and risk of hospitalization has been studied extensively among patients receiving chemotherapy [2–4], including the development of validated predication models [5]. In an analysis of a large prospective randomized study of elderly patients with advanced non-small cell lung cancer (NSCLC) receiving chemotherapy, pre-treatment quality of life as well as ability to perform instrumental activities of daily living were independently associated with prognosis [6]. A prediction model proposed by Hurria et al. for older adults receiving chemotherapy, which utilizes 11 risk factors including geriatric and oncologic frailty measures, was found to be predictive of grade 3–5 toxicity while KPS was not [7]. Nie et all have shown that this prediction model can be applied specifically in the lung cancer population to better distinguish risks of chemotherapy toxicity [5].

While these tools are helpful in older adults with metastatic lung cancers planning to receive chemotherapy, further studies are needed to identify parameters associated with risk of hospitalization among lung cancer patients receiving definitive radiation. Additionally, a better understanding of the impact of hospitalization on oncologic outcomes in these patients is needed. Avoiding unnecessary and often costly hospitalizations [8] is beneficial to both patients, who may suffer from resulting treatment breaks and decreased quality of life, as well as to institutions, who are under increasing pressure to provide cost-effective care. Risk stratification to predict for cancer or treatment related hospitalization is vital for early, aggressive intervention and ultimately prevention.

Extensive pre-treatment assessments have been studied to prognosticate and predict toxicity among geriatric oncology patients [9, 10]. However, the comprehensive nature of these assessments is often their biggest limitation, because clinical practice requires simple, concise and easily measured prediction variables. On the other hand, while performance status, such as the Karnofsky Performance Status (KPS) scale and Eastern Cooperative Oncology Group (ECOG) scale, are frequently assessed by providers, they have not always been shown to be predictive of toxicity [5, 7]. This study aimed to characterize the rate of hospitalization among lung cancer patients receiving definitive intent radiation. We hypothesize that there are baseline clinical markers of frailty in lung cancer patients receiving definitive-intent radiation which can predict risk of hospitalization in order to develop a tool for early identification of patients at risk for cancer or treatment related hospitalization.

## Methods

### Patient selection

Patients treated with thoracic radiation for primary lung cancer between January 2008 and September 2018 at a single tertiary academic institution were identified using institutional registries. Patients planned with conventionally fractionated (1.5–2 Gy per fraction) thoracic radiation to a total dose ≥45 Gy with or without concurrent chemotherapy were included in the cohort. Patients with localized stage I tumors ineligible to receive surgery or local ablative radiotherapy were included in the cohort if they were treated with conventionally fractionated RT. Patients with both non-small cell lung cancer (NSCLC) and small cell lung cancer (SCLC) histology were included. Clinical data was abstracted from the medical record in an IRB-approved database.

### Patient treatment

Patient stage at the time of conventionally fractionated definitive treatment was recorded using the American Joint Committee on Cancer (AJCC) 7th edition staging guidelines. Patients with a prior diagnosis of lung cancer who were treated with definitively dosed RT for isolated local recurrence in the lung or mediastinum were staged as recurrence. Patients with metastatic disease treated with definitively dosed thoracic RT for oligoprogression, oligometastatic disease or consolidative intent were also included. If systemic therapy was delivered with radiation therapy, weekly dosing of carboplatin AUC 2 and paclitaxel was considered a concurrent sensitizing regimen [11, 12]. All other concurrent regimens were considered full dose regimens.

### Baseline characteristics

Baseline characteristics, including age, gender, race, marital status, histology, and stage were all recorded. Specific baseline frailty markers at the start of radiation included: Charlson comorbidity index (CCI), ECOG performance status, patient reported unintentional weight loss, body mass index (BMI), hemoglobin, creatinine, and albumin. Baseline weight/height and lab data were included if collected within 30 days prior to the start of radiation therapy and 14 days after start of RT. If multiple measurements fell within the acceptable time range, the value closest to the start of RT was used.

### Study end points

All unplanned cancer-related hospitalizations during radiation or within 30 days of completing radiation therapy were recorded. Hospitalizations were considered cancer-related if they could be attributable to the disease and/or the treatment. Overall survival (OS) was calculated from 30 days after completing RT to date of death or last follow up in order to assess the relationship between cancer-related hospitalization and survival.

### Statistical analysis

Univariable logistic regression was used to study association of baseline demographic and clinical characteristics and cancer-related hospitalization. Least absolute shrinkage and selection operator (LASSO) method was used to select features that were most significant and build a regression model including selected variables. Tuning parameter of LASSO method was selected to minimize the cross-validation error. Univariable poisson regression were used to examine associations of the number of cancer-related hospitalization with baseline variables. LASSO method with cross-validations was used to select features for multivariable Poisson regression model. A nomogram predicting for cancer-related hospitalization was formulated using LASSO method for selecting variables among the clinical frailty markers, which included CCI, ECOG, unintentional weight loss, BMI, hemoglobin, creatinine, and albumin. Using the same frailty markers selected for in the nomogram, Chi-Square test was used to compare number of risk factors with incidence of hospitalization.

Kaplan-Meier curves were used to estimate survival and univariable Cox proportional-hazards model was used to compare associations of overall survival with demographic and clinical characteristics. LASSO method with cross-validations was used to select features for multivariable Cox proportional-hazards models. All statistical tests were two-sided, and statistical significance was set at *P* ≤ 0.05. Statistical analysis was performed using R version 3.5.3 (R Foundation for Statistical Computing) The package ‘glmnet’ was used for LASSO logistic regression model. The ‘rms’ package was used for prediction nomogram.

## Results

A total of 270 patients planned for conventionally fractionated definitive doses (≥45 Gy) of RT were identified. The baseline patient, tumor and treatment characteristics are summarized in Table 1. Median age was 66.6 years (range 31–88 years), 50.4% were male (*n* = 136), and 60.4% were partnered (*n* = 163). 62.2% of patients were Caucasian (*n* = 162) and 28.1% were black (*n* = 76). The majority of patients were treated for newly diagnosed stage I-III disease (76.3%, *n* = 206) with 3.7% stage I (*n* = 10), 9.3% stage II (*n* = 25) and 63.3% stage III (*n* = 171); 11.9% had metastatic disease (*n* = 32) and 11.9% had treatment for recurrent lung cancer (n = 32) at the time of radiation. The most common histology was adenocarcinoma (53.3%, *n* = 144), followed by squamous cell (32.6%, *n* = 88), other NSCLC histology (10%, *n* = 27), and then small cell (4.1%, *n* = 11).

**Table 1:** Baseline variables and risk of hospitalization

Characteristic	*N* = 270, n (%)	OR (95% CI)	*P*-value
Patient Characteristics			
Age^a^ (years)			
Median	66.6	1.0 (1.0–1.0)	0.80
Range	31–88		
Gender			
Female (reference)	134 (49.6)		
Male	136 (50.4)	1.1 (0.6–2.1)	0.69
Race			
Black (reference)	76 (28.1)		
Caucasian	168 (62.2)	1.4 (0.7–2.8)	0.39
Other	26 (9.6)	0.10 (0.01–1.8)	0.12
Marital Status			
Partnered (reference)	163 (60.4)		
Un-partnered	107 (39.6)	0.8 (0.4–1.5)	0.41
Tumor Characteristics			
Stage Summary			
I-III (reference)	206 (76.3)		
IV	32 (11.9)	0.9 (0.3–2.4)	0.79
Loco-regional recurrence	32 (11.9)	0.7 (0.2–2.0)	0.50
Histology			
Adenocarcinoma (reference)	144 (53.3)		
Squamous cell carcinoma	88 (32.6)	1.9 (1.0–3.8)	0.06
Small cell carcinoma	11 (4.1)	1.7 (0.4–7.8)	0.50
Other	27 (10)	2.0 (0.7–5.4)	0.12
Treatment Characteristics			
Concurrent chemo summary			
Full Dose (reference)	141(52.2)		
Sensitizing	93 (34.4)	1.0 (0.5–1.9)	0.89
No chemo	36 (13.3)	0.4 (0.1–1.4)	0.17
Baseline Frailty Markers			
ECOG PS^a^	*N* = 257		
Median	1	1.7 (1.0–2.9)	0.07
Range	0–3		
CCI^a^	N = 270		
Median	5	1.0 (0.9–1.2)	0.73
Range	2–16		
Patient reported weight loss	*N* = 269		
No (reference)	100 (37.2)		
Yes	169 (62.8)	1.0 (0.5–1.8)	0.90
BMI (kg/m^2^)	*N* = 270		
> 20 (reference)	244 (90.4)		
≤ 20	26 (9.6)	1.9 (0.8–4.9)	0.16
Hemoglobin (g/dL)	*N* = 259		
> 10 (reference)	233 (90.0)		
≤ 10	26 (10.0)	3.3 (1.4–7.9)	0.01
Creatinine (mg/dL)	*N* = 258		
≤ 1.1 (reference)	206 (79.8)		
> 1.1	52 (20.2)	1.1 (0.5–2.3)	0.91
Albumin^b^ (g/dL)	*N* = 254		
Median	3.7	3.1 (1.6–5.9)	< 0.01
Range	2.3–4.8		

^a^ Odds ratio corresponds to 1 point increase in age, ECOG, and CCI

^b^ Odds ratio corresponds to 1 g/dL decrease in albumin

*OR* odds ratio; *ECOG PS* Eastern Cooperative Oncology Group performance status; *CCI* Charlson Comorbidity Index; *BMI* body mass index

86.7% (*n* = 234) of patients received concurrent chemotherapy, of which 60.3% (*n* = 141/234) received full dose chemotherapy and 39.7% (*n* = 93/234) received sensitizing chemotherapy. Median planned treatment dose was 63 Gy (range 45–72 Gy) with a median of 33 fractions (range 21–37)

### Frailty markers

A majority of patients had an ECOG performance status of 0–1 (*n* = 229/257, 89%); 10% (*n* = 26/257) of patients had ECOG of 2 and only 1% (n = 2/257) had an ECOG of 3. Comorbidities were measured using the Charlson Comorbidity Index (CCI) with a median score of 5 (range 2–16). A majority of patients did not report weight loss at the time of consultation or start of treatment (63%, *n* = 169). Median baseline BMI was 25.8 mg/m^2^ with a range of 15.0 to 51.5 mg/m^2^. Other baseline labs evaluated included hemoglobin (median 12.5 g/dL, range 7.8–18.2), creatinine (median 0.9 mg/dL, 0.3–4.4), and albumin (median 3.7 g/dL, 2.3–4.8).

### Cancer-related hospitalization

Among the population, there was a 17% (*n* = 47/270) rate of cancer-related hospitalization with 21% of those hospitalized (*n* = 10/47) having > 1 hospitalization within 30 days of completing RT. Hospitalizations ranged from 1 day after the start of treatment to 24 days after completing treatment with a median admission date of 31 days after starting treatment (Interquartile range (IQR): 16–47 days after start of RT). Univariable analysis of baseline variables associated with hospitalization are outlined in Table 1. On univariable analysis, hemoglobin of ≤10 was associated with 3 times higher risk of hospitalization compared to hemoglobin > 10 (odds ratio (OR) 3.3, 95% confidence interval (CI) 1.4–7.9, *P* = 0.01). Lower albumin was also associated with an increased risk of hospitalization. For each 1 g/dL baseline drop in albumin, there was a 3 times higher risk of hospitalization (OR 3.1, 95% CI 1.6–5.9, *P* = 0.001). Baseline variables and frailty markers associated with > 1 hospitalization are shown in Supplemental Table 1. On univariable analysis, lower baseline albumin with associated with a higher number of hospitalizations (coefficient 2.1, 95% CI 1.3–3.3, *P* = 0.002) as was hemoglobin ≤10 (coefficient 3.1, 95% CI 1.7–5.4, *P* < 0.001), BMI ≤ 20 (coefficient 2.2, 95% CI 1.2–4.1, *p* = 0.02), and squamous histology (coefficient 1.8, 95% CI 1.1–3.2, *p* = 0.03).

The variables predicting for any hospitalization as well as number of hospitalizations (i.e. > 1 hospitalization) selected in multivariable modeling are shown in Table 2. On multivariable analysis, lower albumin and hemoglobin ≤10 remained statistically significant. Hemoglobin ≤10 was associated with 2.4 times higher risk of hospitalization (95% CI 0.8–7.1, *P* = 0.11) and on average, 2.7 more hospitalizations than having a hemoglobin > 10 (95% CI 1.3–5.4, *P* = 0.01). Each 1 g/dL drop in albumin was associated with a 2.4 times higher risk of hospitalization (95% CI 1.2–5.0, *P* = 0.01) as well as increased number of hospitalizations (coefficient 1.5, 95% CI 0.9–1.5, *P* = 0.11) after adjusting for other baseline patient, tumor, and treatment related variables.

**Table 2:** Multivariable model for predictors of hospitalization

	Any Hospitalization		Multiple Hospitalizations	
Variable	OR (95% CI)	*P*-value	Coefficient (95% CI)	*P*-value
Race				
Black (reference)				
Caucasian	1.4 (0.6–3.4)	0.46	1.7 (0.8–3.6)	0.13
Other	0.1 (0.01–2.1)	0.15	0.1 (0.01–2.4)	0.17
Marital Status				
Partnered (reference)				
Un-partnered	0.6 (0.3–1.3)	0.16	0.6 (0.3–1.2)	0.13
ECOG PS ^a^	1.6 (0.9–2.9)	0.15	1.4 (0.9–2.2)	0.11
Hemoglobin (g/dL)				
> 10 (reference)				
≤ 10	2.4 (0.8–7.1)	0.11	2.7 (1.3–5.4)	0.01
Albumin ^b^ (g/dL)	2.4 (1.2–5.0)	0.01	1.5 (0.9–1.5)	0.11

^a^ Odds ratio corresponds to 1 point increase in ECOG

^b^ Odds ratio corresponds to 1 g/dL decrease in albumin

OR, odds ratio; ECOG PS, Eastern Cooperative Oncology Group performance status

**Fig. 1:**
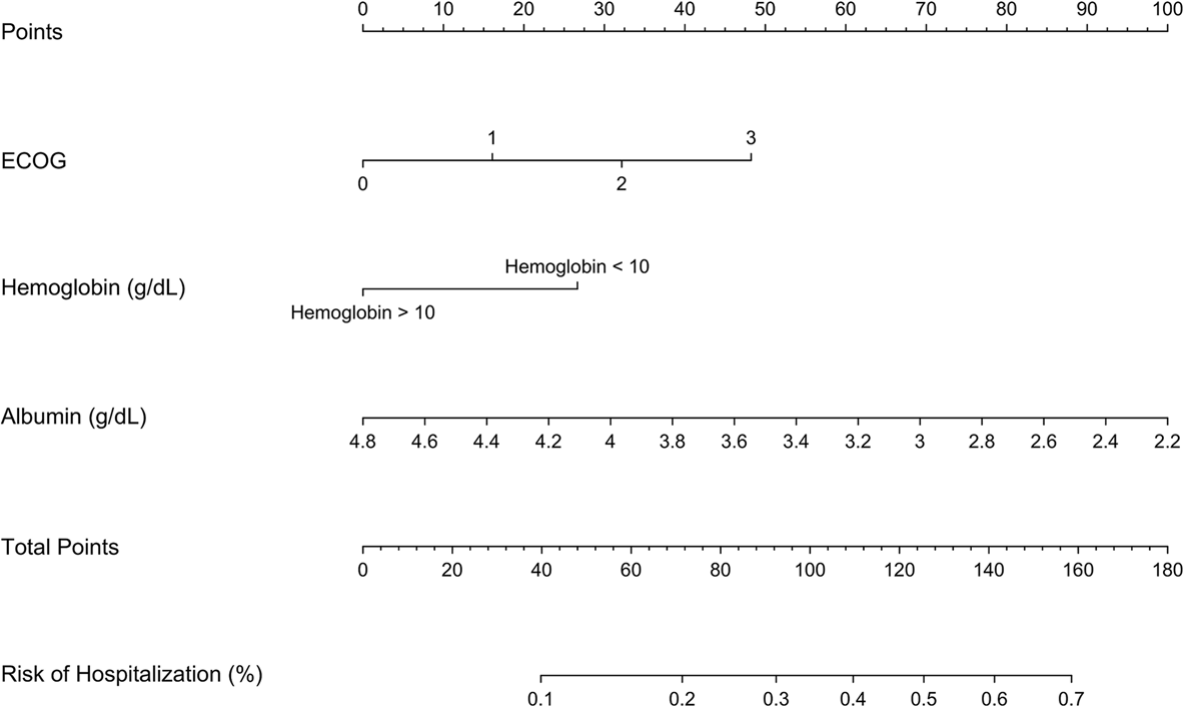
Nomogram predicting risk of hospitalization. Baseline clinical variables, including Eastern Cooperative Oncology Group (ECOG) scale (0–3), hemoglobin (≤ 10 g/dL or > 10 g/dL), and albumin (g/dL), are used in a nomogram to predict risk of hospitalization during or within 30 days of completing thoracic radiation

Figure 1 shows a nomogram model predicting the risk of cancer-related hospitalization during or within 30 days of treatment. This model includes ECOG, hemoglobin, and albumin, which were the variables selected on multivariable modeling from amongst the baseline clinical frailty markers. As an example, this model would predict a patient with ECOG 2, hemoglobin 9 g/dL, and albumin of 3 g/dL, would have around a 55% risk of hospitalization.

**Fig. 2:**
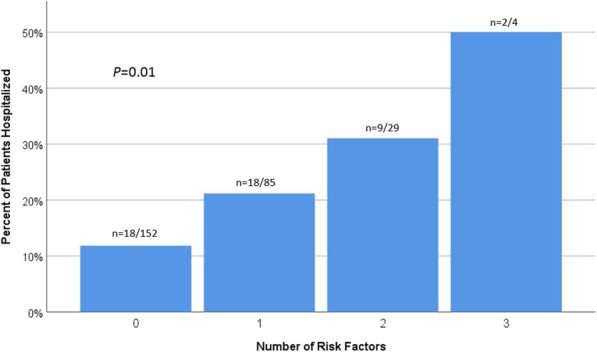
Number of risk factors and incidence of hospitalization. Risk factors included: Eastern Cooperative Oncology Group (ECOG) scale ≥2, hemoglobin ≤10 g/dL, and albumin ≤3.5 g/dL. Significant association between number of risk factors and rate of hospitalization (*X*^2^ (3)=10.8, *P* = 0.01). As the number of risk factors increased from 0 to 3, the percentage of patients hospitalized increased from 11.8, to 21.1%, to 31%, and to 50%, respectively

Figure 2 shows the relationship between number of risk factors and rate of hospitalization. Risk factors included the same frailty markers as above but as categorical variables: ECOG ≥2, hemoglobin ≤10, and albumin ≤3.5. There was a significant association between number of risk factors and rate of hospitalization (*X*^2^ (3)=10.8, *P* = 0.01). As the number of risk factors increased from 0 to 3, the percentage of patients hospitalized increased from 11.8% (*n* = 18/152), to 21.1% (n = 18/85), to 31% (*n* = 9/29), and to 50% (*n* = 2/4), respectively.

### Survival

**Fig. 3:**
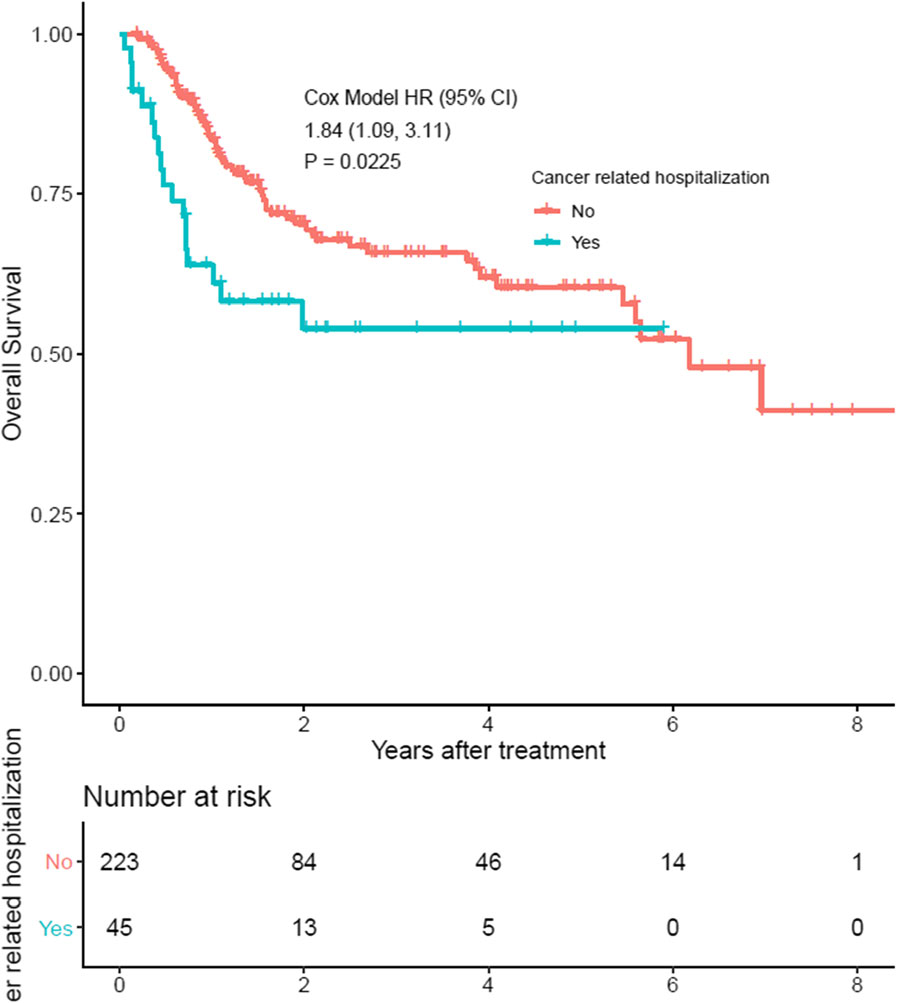
Kaplan-Meier Overall Survival. Kaplan-Meier plot of overall survival among those who were and were not hospitalized during or within 30 days of radiation

The median follow up was 17 months (range 0.6–102 months). Two and three year overall survival for the cohort was 68 and 64%, respectively. Table 3 shows univariable and multivariable analysis of overall survival. On unadjusted modeling, baseline factors associated with increased mortality included: increasing age (Hazard Ratio (HR) 1.0, 95% CI: 1.0–1.1, *P* = 0.02), male gender (HR 1.7, 95% CI: 1.1–2.7, *P* = 0.01), squamous histology (HR 1.6, 95% CI: 1.0–2.6, *P* = 0.04), sensitizing compared to full dose chemotherapy (HR 2.0, 95% CI: 1.3–3.2, *P* = 0.004), increasing ECOG (HR 1.7, 95% CI: 1.2–2.4, *P* = 0.01), increasing CCI (HR 1.2, 95% CI: 1.1–1.3, *P* < 0.001), BMI ≤ 20 (HR 2.7, 95% CI: 1.5–4.8, *P* = 0.001), hemoglobin ≤10 (HR 2.5, 95% CI: 1.4–4.5, *P* = 0.002), and lower albumin (HR 3.2, 95% CI: 1.0–5.0, *P* < 0.001). On unadjusted modeling, the risk of death was 1.8 times higher among those hospitalized during treatment compared to those who were not (95% CI 1.1–3.1, *P* = 0.02). Kaplan-Meier curves for overall survival of those who were hospitalized and those who were not hospitalized are shown in Fig. 3. The two year overall survival was 54% among those who were hospitalized compared to 70% among those who were not hospitalized.

**Table 3:** Predictors of mortality

	Univariable		Multivariable	
Variable	HR (95% CI)	*P*-value	HR (95% CI)	*P*-value
Patient Characteristics				
Age ^a^ (years)	1.0 (1.0–1.1)	0.02	1.0 (1.0–1.1)	0.19
Gender				
Female (reference)				
Male	1.7 (1.1–2.7)	0.01	2.1 (1.3–3.4)	< 0.01
Race				
Black (reference)				
Caucasian	0.9 (0.6–1.4)	0.54	–	
Other	0.6 (0.2–1.4)	0.21	–	
Marital Status				
Partnered (reference)				
Un-partnered	1.1 (0.7–1.7)	0.56	–	
Tumor Characteristics				
Stage Summary				
I-III (reference)				
IV	1.6 (0.8–2.9)	0.17	–	
Loco-regional recurrence	1.2 (0.7–2.3)	0.53	–	
Histology				
Adenocarcinoma (reference)				
Squamous cell carcinoma	1.6 (1.0–2.6)	0.04	–	
Small cell carcinoma	2.3 (0.9–5.6)	0.08	–	
Other	1.6 (0.8–3.5)	0.21	–	
Treatment Characteristics				
Cancer related hospitalization				
No (reference)				
Yes	1.8 (1.1–3.1)	0.02	1.8 (1.0–3.1)	0.04
Relative weight loss during treatment				
≤ 5% weight loss (reference)				
> 5% weight loss	1.5 (1.0–2.4)	0.08	–	
Concurrent chemo summary				
Full Dose (reference)				
Sensitizing	2.0 (1.3–3.2)	< 0.01	1.4 (0.8–2.5)	0.21
No chemo	1.4 (0.7–2.6)	0.32	1.3 (0.5–3.3)	0.56
Baseline Frailty Markers				
ECOG PS^a^	1.7 (1.2–2.4)	0.01	1.6 (1.1–2.4)	0.02
CCI^a^	1.2 (1.1–1.3)	< 0.01	1.1 (1.0–1.2)	0.25
Patient reported weight loss				
No (reference)				
Yes	1.5 (1.0–2.3)	0.08	–	
BMI (kg/m^2^)				
> 20 (reference)				
≤ 20	2.7 (1.5–4.8)	< 0.01	3.2 (1.7–6.1)	< 0.01
Hemoglobin (g/dL)				
> 10 (reference)				
≤ 10	2.5 (1.4–4.5)	< 0.01	1.2 (0.6–2.4)	0.65
Creatinine (g/dL)				
≤ 1.1 (reference)				
> 1.1	1.4 (0.9–2.4)	0.14	–	
Albumin^b^ (g/dL)	3.2 (1.0–5.0)	< 0.01	2.9 (1.7–4.5)	< 0.01

^a^ Hazard ratio corresponds to 1 point increase in age, ECOG, and CCI

^b^ Hazard ratio corresponds to 1 g/dL decrease in albumin

*HR* hazard ratio; *ECOG PS* Eastern Cooperative Oncology Group performance status; *CCI* Charlson Comorbidity Index; *BMI* body mass index

After adjusting for potential cofounders using multivariable modeling, the variables which were still associated with increased mortality included: male gender (HR 2.1, 95% CI: 1.3–3.4, *P* = 0.004), increasing ECOG (HR 1.6, 95% CI: 1.1–2.4, *P* = 0.02), BMI ≤20 (HR 3.2, 95% CI: 1.7–6.1, *P* = 0.001), and decreasing albumin (HR 2.9, 95% CI: 1.7–4.5, *P* < 0.001). After controlling for other baseline variables including age, comorbidities, concurrent chemotherapy, and baseline hemoglobin, cancer-related hospitalization was still associated with 1.8 times increased risk of death (95% CI: 1.02–3.1, *P* = 0.04).

## Discussion

Among those receiving definitively dosed thoracic radiation, there was a 17% rate of cancer-related hospitalization, of which 21% of those hospitalized had > 1 hospitalization within 30 days of completing RT. Early identification of patients most at risk can identify those who are most likely benefit from close monitoring and early interventions such as out-patient hydration, nutritional support, and/or symptom management. In this study, we have identified specific baseline factors, including low albumin and low hemoglobin, which are associated with increased risk and increased number of hospitalizations, respectively.

Past studied have shown that pre-treatment hemoglobin/hematocrit and albumin are prognostic factors for outcomes among those with cancer and specifically, those with lung cancer [13–15]. Our study adds to this body of literature by further validating this observation in a previously unstudied population, those with lung cancer receiving definitive radiation.

Other comprehensive models predicting for toxicity and outcomes have been validated for patients receiving systemic therapy, but due to their extensive nature covering numerous domains, may be difficult to apply in clinical practice [16, 17]. Using select baseline clinical factors, including ECOG, hemoglobin, and albumin, we have created a concise nomogram to predict for cancer-related hospitalization among those receiving definitive doses of thoracic radiation. This model uses baseline clinical variables which can be easily obtained by clinicians at the start of treatment and does not require additional patient reported assessments of frailty. To our knowledge, this is the first proposed prediction tool for cancer-related hospitalization in this population.

The 2 year overall survival of our cohort is similar to historical controls [18, 19]. In the randomized phase III PACIFIC trial, 24 month overall survival of patients receiving chemoradiation followed by Durvalumab was 66% compared to a 24 month OS of 68% in our cohort [19]. Our survival analysis demonstrates the significance of cancer-related hospitalization and its potential effects on mortality. We identified a number of statistically significant variables associated with increased mortality on multivariable modeling including male gender, lower performance status, BMI ≤20, low albumin, as well as cancer-related hospitalization. This suggests that even after adjusting for other baseline patient and treatment characteristics, being hospitalized during or within 30 days of completing radiation increases the risk of early mortality.

Past studies have shown that the actual risk of hospitalization among patients undergoing chemotherapy may be as high as 40–50%, especially among the elderly or those with metastatic disease [20, 21]. In a retrospective review of 1116 patients receiving both curative and palliative radiation, the risk of hospitalization during or within 90 days of completing radiation was 20% among all patients and 25% among those being treated for lung cancer [22]. In comparison, we observed a 17% rate of hospitalization in our cohort, which consisted of the “best players” in that we included all age groups and stages and only those who were planned for definitively dosed radiation with a majority (87%) receiving concurrent chemotherapy. In the modern health care era of rising health care costs, payments are being tailored to reflect quality care, with particular focus on hospital readmission rates [23, 24]. Patients undergoing radiation therapy are in the unique situation of having frequent clinical assessments by members of the health care team as they receive daily treatment often for multiple weeks at a time. Providing additional outpatient supportive care to those at-risk for hospitalization and readmission has the potential to directly benefit both the patient and the hospital system.

Due to the retrospective nature of this study, our ability to include patient reported variables related to function, cognition, and physical performance, which may be better predictors of frailty and ultimately hospitalization, was limited. Additionally, the patient population was heterogeneous in regards to disease status and treatment paradigm. This, in conjunction with our limited sample size, limited our ability to evaluate all potentially important variables such as initially stage, radiation biologically effective dose, or specific chemotherapy regimen. There may also be dynamic variables which change over the course of treatment and have their own predictive value in identifying those at risk. However, the focus of this study was on utilizing simple baseline clinical objective measures, which are regularly obtained and recorded. This has resulted in a robust and parsimonious prediction tool. Lastly, since we are proposing a predictive tool which may have applicability in a clinical setting, validation among multi-institutional or a prospective cohort is necessary before it can be generalized to a specific patient population.

## Conclusions

The results of this study not only highlight the prevalence of hospitalization and impact on survival but also identify certain baseline variables which are associated with an increased risk of hospitalization. Using these data, prospective longitudinal studies can be designed to further validate the predictive model of cancer-related hospitalization among those receiving definitively dosed radiation therapy. More importantly, validation of our predictive model will help identify those patients who are most likely to benefit from preventative interventions during treatment to avoid hospitalization and improve outcomes.

## Supplementary information

**Additional file 1: Table S1.** Baseline variables and risk of multiple hospitalizations.

## Data Availability

The datasets generated and/or analyzed during the current study are not publicly available due to patient privacy restrictions put in place by the institutional IRB preventing the action of non-approved data sharing. Data may be available from the first author S. Hazell on reasonable request and subsequent institutional approval.

## Abbreviations

ECOG: Eastern Cooperative Oncology Group
BMI: Body mass index
RT: Radiation
NSCLC: Non-small cell lung cancer
KPS: Karnofsky Performance Status
SCLC: Small cell lung cancer
AJCC: American Joint Committee on Cancer
CCI: Charlson comorbidity index
OS: Overall survival
LASSO: Least absolute shrinkage and selection operator IQR Interquartile range
OR: Odds ratio
CI: Confidence interval
