# Supplementary material for: Hospitalization and definitive radiotherapy in lung cancer: incidence, risk factors and survival impact
Source: BMC Cancer. 2020 Apr 19;20:334. doi: 10.1186/s12885-020-06843-z (PMC7169027; doi:10.1186/s12885-020-06843-z)
Supplement: Supplementary file 1 — Additional file 1: Table S1. Baseline variables and risk of multiple hospitalizations. [file 12885_2020_6843_MOESM1_ESM.docx]

| Supplemental Table 1: Baseline variables and risk of multiple hospitalizations | | |
| --- | --- | --- |
| Characteristic | Coefficient (95% CI) | P-value |
| Patient Characteristics |  |  |
| Age^a^ (years) | 1.0 (0.9-1.0) | 0.53 |
| Gender |  |  |
| Female (reference) |  |  |
| Male | 1.4 (0.8-2.3) | 0.22 |
| Race |  |  |
| Black (reference) |  |  |
| Caucasian | 1.8 (0.9-3.3) | 0.08 |
| Other | 0.1 (0.0-2.0) | 0.14 |
| Marital Status |  |  |
| Partnered (reference) |  |  |
| Un-partnered | 0.7 (0.4-1.2) | 0.22 |
| Tumor Characteristics |  |  |
| Stage Summary |  |  |
| I-III (reference) |  |  |
| IV | 1.2 (0.6-2.4) | 0.70 |
| Loco-regional recurrence | 0.8 (0.3-1.8) | 0.51 |
| Histology |  |  |
| Adenocarcinoma (reference) |  |  |
| Squamous cell carcinoma | 1.8 (1.1-3.2) | 0.03 |
| Small cell carcinoma | 1.3 (0.4-4.9) | 0.66 |
| Other | 1.6 (0.7-3.7) | 0.24 |
| Treatment Characteristics |  |  |
| Concurrent chemo summary |  |  |
| Full Dose (reference) |  |  |
| Sensitizing | 1.0 (0.6-1.7) | 0.94 |
| No chemo | 0.6 (0.3-1.6) | 0.34 |
| Baseline Frailty Markers |  |  |
| ECOG PS^a^ | 1.5 (1.0-2.3) | 0.07 |
| CCI^a^ | 1.0 (0.9-1.1) | 0.75 |
| Patient reported weight loss |  |  |
| No (reference) |  |  |
| Yes | 1.1 (0.7-1.9) | 0.63 |
| BMI (kg/m^2^) |  |  |
| >20 (reference) |  |  |
| ≤20 | 2.2 (1.2-4.1) | 0.02 |
| Hemoglobin (g/dL) |  |  |
| >10 (reference) |  |  |
| ≤10 | 3.1 (1.7-5.4) | <0.001 |
| Creatinine (mg/dL) |  |  |
| ≤1.1 (reference) |  |  |
| >1.1 | 1.4 (0.8-2.4) | 0.31 |
| Albumin^b^ (g/dL) | 2.1 (1.3-3.3) | 0.002 |

^a^ Coefficient corresponds to 1 point increase in age, ECOG, and CCI

^b^ Coefficient corresponds to 1 g/dL decrease in baseline albumin

ECOG PS, Eastern Cooperative Oncology Group performance status; CCI, Charlson Comorbidity Index; BMI, body mass index
